# Targeted Disruption of miR-17-92 Impairs Mouse Spermatogenesis by Activating mTOR Signaling Pathway

**DOI:** 10.1097/MD.0000000000002713

**Published:** 2016-02-18

**Authors:** Raoying Xie, Xiaolin Lin, Tao Du, Kang Xu, Hongfen Shen, Fang Wei, Weichao Hao, Taoyan Lin, Xia Lin, Yujuan Qin, Huiyan Wang, Lin Chen, Sheng Yang, Jie Yang, Xiaoxiang Rong, Kaitai Yao, Dong Xiao, Junshuang Jia, Yan Sun

**Affiliations:** From the Cancer Research Institute, Southern Medical University (RX, XL, HS, FW, WH, TL, XL, YQ, HW, LC, SY, JY, KY, DX, JJ); Institute of Comparative Medicine and Laboratory Animal Center, Southern Medical University (RX, DX); Zhongshan School of Medicine, Sun Yat-sen University (YS); Department of Endocrinology, The Second Affiliated Hospital, Guangzhou Medical University (TD); Department of General Surgery, Sun Yat-sen Memorial Hospital, Sun Yat-sen University, Guangzhou (KX); Department of Chemoradiotherapy, The First Affiliated Hospital of Wenzhou Medical University, Wenzhou (RX); Guangdong Provincial Key Laboratory of Malignant Tumor Epigenetics and Gene Regulation, Sun Yat-Sen Memorial Hospital, Sun Yat-Sen University (KX); Department of Oncology, Nanfang Hospital, Southern Medical University (XR); and Guangzhou Digestive Disease Center, Guangzhou First People's Hospital, Guangzhou Medical University, Guangzhou, China (FW).

## Abstract

The miR-17-92 cluster and its 6 different mature microRNAs, including miR-17, miR-18a, miR-19a, miR-20a, miR-19b-1, and miR-92a, play important roles in embryo development, immune system, kidney and heart development, adipose differentiation, aging, and tumorigenicity. Currently, increasing evidence indicates that some members of miR-17-92 cluster may be critical players in spermatogenesis, including miR-17, miR-18a, and miR-20a. However, the roles and underlying mechanisms of miR-17-92 in spermatogenesis remain largely unknown. Our results showed that the targeted disruption of miR-17-92 in the testes of adult mice resulted in severe testicular atrophy, empty seminiferous tubules, and depressed sperm production. This phenotype is partly because of the reduced number of spermatogonia and spermatogonial stem cells, and the significantly increased germ cell apoptosis in the testes of miR-17-92-deficient mice. In addition, overactivation of the mammalian target of rapamycin signaling pathway and upregulation of the pro-apoptotic protein Bim, Stat3, c-Kit, and Socs3 were also observed in miR-17-92-deficient mouse testes, which might be, at least partially if not all, responsible for the aforementioned phenotypic changes in mutant testes. Taken together, these findings suggest that miR-17-92 is essential for normal spermatogenesis in mice.

## Abstract 2

Supplemental Digital Content is available in the text

## INTRODUCTION

Spermatogenesis is a complicated process that involves numerous molecules and pathways. Dysregulation at any stages of spermatogenesis may cause infertility.^1^ Currently, the infertility as a public health problem has received widespread attention. One in every 4 couples in developing countries has been found to be affected by infertility in a WHO study. A large part of male infertility is suffering from severe oligozoospermia or azoospermia. However, the underlying molecular mechanisms remain largely unknown.

MicroRNAs (miRNAs), a class of small noncoding RNA molecules, function by regulating gene expression via degradation or translational inhibition of their target mRNAs, and thus participate in a wide variety of physiological and pathological processes, including development, cell proliferation, differentiation and apoptosis, metabolism, cancer, etc.^2,3^ In addition, miRNAs are required for the proliferation of primordial germ cells (PGCs) and spermatogonia.^4^ The miR-17-92 gene cluster encodes 6 miRNAs of 4 miRNA families: the miR-17 family including miR-17 and miR-20a, the miR-18 family (miR-18a), the miR-19 family (miR-19a and miR-19b-1), and the miR-92 family.^5^ As a typical multifunctional gene cluster, miR-17-92 plays crucial roles in various physiological and pathological processes, such as immune system,^6,7^ development,^8^ polycystic kidney disease,^9,10^ carotid artery restenosis,^11^ idiopathic pulmonary fibrosis,^12^ heart development,^13^ adipocyte differentiation,^14^ and cancer.^5,15^ On the other hand, there are several lines of evidence that miR-17-92 is involved in spermatogenesis.^16–19^ The in situ hybridization analysis on adult testes revealed that the miR-17 is highly expressed in early stages of germ cells and greatly decreased as germ cells mature, and miR-20a is mainly detected in the spermatogonia and preleptotene spermatocytes.^15^ miR-20 is preferentially expressed in mouse spermatogonial stem cells (SSCs) and essential for self-renewal of SSCs.^19^ miR-18 displays a cell-type-specific expression, with highest intensity in the spermatocytes.^18^ Moreover, the in situ hybridization analysis shows that pri-miR-17-5p expression in human testes is lowest in a subset of spermatogonia and early spermatocytes close to the seminiferous tubule edge, and is increased with germ cell maturation proceeding toward lumen of the seminiferous tubule.^17^ More importantly, several studies have reported that miRNAs, including the miR-17-92 cluster,^20^ play critical roles in nonobstructive azoospermia. The aforementioned findings suggest that miR-17-92 might play a significant role in spermatogenesis, which remains to be fully characterized.

To address this issue, we generated mice with miR-17-92 deletion in adult mouse testes by using the Cre/loxP system. miR-17-92-deficient mice displayed the smaller size and the lighter weight of testes, empty seminiferous tubules, and the reduced sperm number. Interestingly, spermatogonia and SSCs were found to be lost and apoptotic germ cells were detected in miR-17-92-deficient mouse testes. Furthermore, we also showed the dysregulated mTOR signaling and the upregulation of the proapoptotic protein Bim, Stat3, c-Kit, and Socs3, which are at least in part if not all contributed to the phenotypes observed in the testes of miR-17-92-deficient mice.

## METHODS

### Mice

The heterozygous hUb-Cre-ERT2 mice [129S.Cg-Tg(UBC-cre/ESR1)1Ejb/J; Stock Number: 007179]^21^ and the homozygous R26R reporter mice (B6;129S-Gt(ROSA)26sor/JNju; Stock Number: 002073)^22^ were purchased from Model Animal Research Center of Nanjing University, China. hUb-CreERT2 mice were generated through lentitransgenesis using a lentivirus that expresses the Cre-ERT2 from the human ubiquitin C promoter.^21^ The miR-17-92fl^/fl^ mice (B6;129S4-Mirn17-92tm1.1Tyj/J: Stock Number: 008459)^23^ were obtained from the Jackson Laboratory.

All animals care and experimentation were performed according to the Study and Ethical Guidelines for Animal Care, handling and termination established by the Subcommittee of Southern Medical University on laboratory animal care. The presented work was approved by the ethical committee of Southern Medical University and is covered by Chinese animal husbandary legislation.

### Cell Culture

293T and GC-1 cell lines are available from the Cancer Research Institute of Southern Medical University. GC-1 cell line is a mouse-derived spermatogonia line.^24^ These cell lines used in this study were maintained in Dulbecco's Modified Eagle's Medium with 10% fetal bovine serum (FBS; Biological Industries, Jerusalem, Israel) and incubated at 5% CO_2_ at 37°C.

### Deletion of miR-17-92 in Adult Mice

As shown in Figure S1A, to delete miR-17-92 in adult mice, the miR-17-92fl^/fl^Cre-ERT2 mice were obtained after 2 rounds of mating. Then, the 8-week-old miR-17-92fl^/fl^ Cre-ERT2 mice were treated by intraperitoneal injection of 20 mg/mL tamoxifen in corn oil (0.2 mg/g body weight, once per day for 5 days) to conditionally delete miR-17-92 cluster in adult mice.

### Genotype Analysis by PCR

**FIGURE 1:**
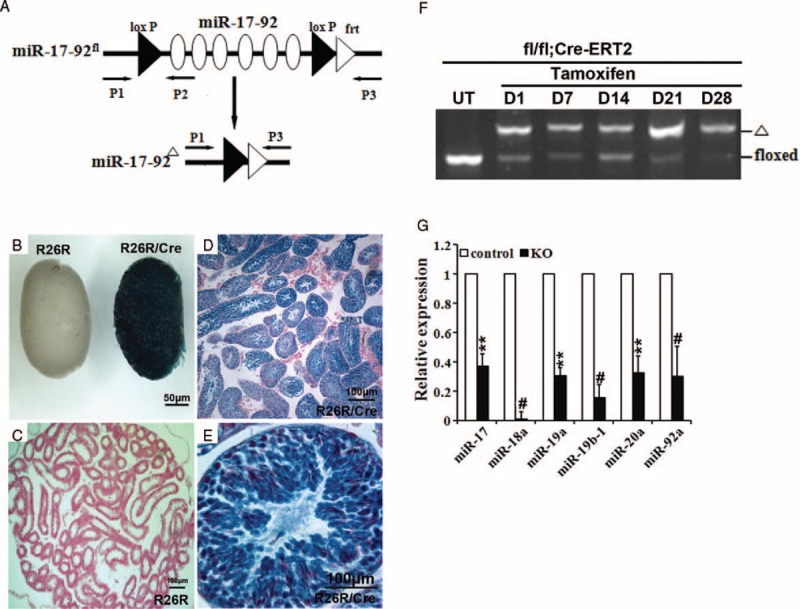
A drug-inducible system to efficiently delete miR-17-92 cluster in adult mouse testes. A, Targeting scheme for the miR-17-92 cluster conditional deletion. B–E, X-Gal staining showing the high efficiency of Cre-mediated recombination in testis of adult R26R/hUb-Cre-ERT2 mice. Eight week old R26R/hUb-Cre-ERT2 mice were treated by intraperitoneal injection of 20 mg/mL tamoxifen in corn oil (0.2 mg/g body weight, once per day for 5 days) to activate the Lac Z expression. F, PCR-based genotyping was performed on genomic DNA from the testes of miR-17-92-deficient mice. UT: untreated with tamoxifen; D1, D7, D14, D21 and D28 represent the 1st, 7th, 14th, 21st, and 28th days after the last tamoxifen injection in miR-17-92fl^/fl^/hUb-Cre-ERT2 mice, respectively. G, qRT-PCR analysis of the expression of the individual components of miR-17-92 gene cluster in testes of miR-17-92 KO mice (n > 5).

To obtain miR-17-92fl^/fl^Cre-ERT2 mice, we identified the Cre gene after the first crossing, and then we identified homozygous miR-17-92fl^/fl^ and Cre-positive mice after the second crossing. Genomic DNA was prepared from mouse tail or testis tissue using the TIANamp Genmic DNA Kit (Tiangen, China) and according to our previous publications,^25,26^ and the mouse tail DNA was subjected to PCR using primer pair specific for Cre (Table S1) and P1/P2 primer pair (Figure 1A and Table S1) to amplify a 102-bp Cre fragment (Figure S1B and D), and 255 or/and 289-bp fragments (Figure S1C), respectively. Moreover, to assess the knockout efficiency of miR-17-92 in adult testes, the mouse testis tissue DNA was subjected to PCR using P1/P3 primer pair (Figure 1A and Table S1) to amplify 441-bp miR-17-92 mutant fragment.

### Whole-Mount X-Gal Staining and Histology

To evaluate Cre-ERT2 mice-mediated excision efficiency in testis, hUb-Cre-ERT2 mice were mated with a Cre-dependent Lac Z reporter strain (R26R reporter mice)^22^ to generate R26R/Cre-ERT2 mice, while Lac Z expression is activated upon Cre-mediated excision of the floxed CAT gene in R26R/Cre-ERT2 mice. The testes were collected and stained in X-Gal staining solution as described previously.^27^

### RNA Isolation and Quantitative Real-Time PCR (qRT-PCR)

Total RNAs were extracted from testis tissues at D1, D7, D14, D21, and D28 using TRIzol (TaKaRa, Tokyo, Japan) according to the manufacturer's protocol and our previous publications.^26,28,29^ Total RNA was reversely transcribed using the iScript cDNA synthesis kit (BIO-RAD, California, USA). The expression levels of different components of miR-17-92 and the indicated genes were determined using SYBR Green quantitative PCR performed with the Stratagene Mx3005P Real-Time PCR system (Agilent Technologies Inc, California, USA). The primer pairs used for the amplification of the indicated genes were listed in Table S2–4. To measure the levels of miR-17-92 and the indicated gene mRNAs, U6 snRNA and β-actin were used as endogenous control, respectively. The results were calculated by the relative quantification protocol (2^-△△Ct^).

### Histological Analysis and Immunohistochemistry

Testes or epididymides were harvested from miR-17-92 KO and control mice at D1, D7, D14, D21, and D28. The tissues were fixed in 4% paraformaldehyde for 24–72 h, and then dehydrated in 70%, 80%, 95% and absolute ethanol, and embedded in paraffin blocks. Blocks were cut into 5-μm sections. The sections were mounted on slides, deparaffinized in 100% xylene, and rehydrated in a descending ethanol series and water, followed by hematoxylin and eosin staining (H&E staining) according to standard procedures.

Immunohistochemical staining was performed according to the standard streptavidin-peroxidase (SP) method, as previously described.^30^ We prepared histological sections as previously described in H&E staining, and paraffin sections (5 μm) from mouse testes were deparaffinized in 100% xylene and subsequently rehydrated in a descending ethanol series (100%, 90%, 80%, and 70% ethanol) and water according to standard procedures. Heat-induced epitope retrieval was performed using a high-pressure cooker in 10-mM sodium citrate buffer (pH 6.0) for 2 min. The sections were then incubated with 3% hydrogen peroxide in PBS for 10 min to quench the endogenous peroxidase activity and nonspecific antigens were blocked using 1% bovine serum albumin (BSA) for 60 min at room temperature, respectively, followed by incubation with rabbit anti-mouse 4EBP1 antibody (CST, Boston, USA; dilution: 1:400), rabbit anti-mouse p4EBP1 antibody (CST, Boston, USA; dilution: 1:400), rabbit anti-mouse S6 antibody (CST, Boston, USA; dilution:1:100), rabbit anti-mouse pS6 antibody (CST, Boston, USA; dilution:1:200), and mouse antimouse BrdU antibody (GE Healthcare, Massachusetts, USA; dilution: 1:50) overnight at 4°C, respectively. The antibodies and conditions used are summarized in Table S5. The next day, the sections were washed 3 times in PBS, and incubated with streptavidin-conjugated horseradish peroxidase (HRP)-IgG goat antimouse or goat antirabbit secondary antibody for 30 min at room temperature. The antigen–antibody reaction was developed using 3,3-diam-inobenzidine (DAB) as a chromogen substrate, the nuclei were counterstained with hematoxylin.

The mitotic spermatogonia number was determined by bromodeoxyuridine (BrdU) incorporation.^31^ Tissue samples for mitotic index calculation were obtained from male mice intraperitoneally injected with BrdU (Sigma, St. Louis, Missouri, USA) (100 μg/g body weight) once per day for 6 days. After 6 days, the mice were sacrificed and testes were collected. The antibodies to BrdU labeled the mitotic spermatogonia cells. The staining procedure is the same as immunohistochemistry. The index of mitotic spermatogonia number was determined as the average number of BrdU-positive nuclei per seminiferous tubule.

### Sperm Counts and Motility

For each mouse (D1, D7, D14, D21, and D28), whole epididymis and vas deferens were harvested, cut into 2-mm-long pieces, and resuspended in 1 mL of physiological saline containing 0.4% BSA. The pieces and sperm fluid were homogenized at 37°C for 10 min to dissociate the somatic cells. Sperm cells that remained as a monodispersed suspension were counted using a hemocytometer. The sperm cell viability was assessed by microscopy. To assess sperm morphology, we took 10 μL sperm mixture for sperm smear. Then, we stained sperm by using Hematoxylin and samples were observed by microscopy.

### Whole-Mount Immunofluorescence

We dissected testes from capsules and placed them in a dish of cold phosphate-buffered saline (PBS) in an ice slurry. The tubule segments were dissociated from one another and from the interstitial material using microsurgery forceps under a dissecting microscope. The whole-mount staining procedure for seminiferous tubules was described previously.^32^ The tubules were incubated with rabbit antibody against Foxo1 (CST, Boston, USA; 1:50 dilution) in PBS at room temperature overnight, and then washed 3 times with PBS at room temperature. Subsequently, they were incubated with an Alexa Fluor 488-conjugated goat antirabbit antibody (Invitrogen, California, USA; 1:100 dilution) at room temperature for 2 h. After washing with PBS, the tubules were stained for 10 min with DAPI in PBS, and washed briefly in PBS. Finally, tubules were placed on glass slides and mounted in Vectashield (Vector Laboratories, California, USA). All photos were taken with a Nikon A1 confocal microscope with a ×40 water immersion lens using 561-nm HeNe laser (26% full power) and 488-nm Ar laser (15% full power) excitation.

### TUNEL Assay

For terminal deoxynucleotidyl transferase-mediated dUTP-biotin nick-end labeling (TUNEL) analysis, we prepared histological sections as previously described in H&E staining. Formalin-fixed sections were deparaffinized, rehydrated, and pretreated with proteinase K. Apoptotic cells in testis tissues were detected using a TUNEL kit according to the manufacturer's instructions (KeyGEN, KGA704, Nanjing, China).

### Western Blot Analysis

Proteins were extracted from mouse testes. The protocols for Western blot were previously well described.^28,29,33^ The origin and description of all antibodies used in this study are shown in Table S5.

### Luciferase Assay

The luciferase reporter gene vectors (ie, pLuc-Bim-3’-UTR-wt and pLuc-Stat3-3’-UTR-wt) containing the putative miR-20a binding site at the 3’-UTR of Bim or Stat3 mRNA were obtained from Kangbio (Shenzhen, China). 293T and GC-1 cells were seeded in 96-well plate (1 × 10^5^ cells/well) and incubated for 24 h. The pLuc-Bim-3’-UTR-wt or pLuc-Stat3-3’-UTR-wt was cotransfected into 293T or GC-1 cells with miR-20a mimics, mimics control, or miR-20a mimics plus miR-20a inhibitor using Lipofectamine 2000 (Invitrogen, California, USA), respectively. Cells were harvested 48 h after transfection and detected for firefly and Renilla luciferase activities using the Dual-Luciferase Reporter Assay System (Promega, Wisconsin, USA) according to the manufacturer's instructions and our previous publications.^26,28^

### Statistical Analysis

Data were presented as means ± SD of at least 3 independent experiments. SPSS 13.0 software was used for the statistical analysis. The results were analyzed using a 2-tailed Student *t* test. *P* < 0.05 was considered statistically significant (^∗^*P* < 0.05; ^∗∗^*P* < 0.01, ^#^*P* < 0.000).

## RESULTS

### hUb-Cre-ERT2 Mouse Line Efficiently Mediates DNA Recombination in Testes

We first investigated the hUb-Cre-ERT2-mediated excision efficiency in testis by crossing hUb-Cre-ERT2 mice with the R26R reporter mouse line.^22^ Mouse testis heterozygous for both the hUb-Cre-ERT2 and R26R alleles displayed ubiquitous blue staining after whole-mount X-Gal staining (Figure 1B), while the sections of hUb-Cre-ERT2/R26R testis exhibited a high level of X-Gal staining (Figure 1D and E). In summary, these data indicate that hUb-Cre-ERT2-mediated recombination is highly efficient in mouse testis.

### miR-17-92 Gene Cluster Is Efficiently Deleted in Adult Mouse Testes by Using hUb-Cre-ERT2 Mice

If miR-17-92 is knocked out at the early stage of embryo development, the newborns invariably die within minutes after birth.^8^ To investigate the effects of miR-17-92 deletion on mouse spermatogenesis postnatally, we used the aforementioned hUb-Cre-ERT2 mice.^21^

To test the efficiency of Cre recombinase activation, adult miR-17-92^fl/fl^/Cre-ERT2 mice (6–8 weeks of age) were treated once per day for 5 days with tamoxifen (Figure 1F). 1, 7, 14, 21, and 28 days after the final tamoxifen treatment, mice were sacrificed and DNA from testis tissues was analyzed by PCR to quantify the miR-17-92^fl/fl^ and miR-17-92^Δ^ alleles (Figure 1F). Efficient recombination (>70%) was observed in adult testes examined (Figure 1F). In addition to the genomic locus, we analyzed the expression of the individual components of miR-17-92 in adult testes of miR-17-92 KO mice. As shown in Figure 1G, RT-PCR analysis revealed that the expression of the individual components of miR-17-92 was significantly downregulated in adult testes of miR-17-92 KO mice, compared with control testes. Thus, the hUb-Cre-ERT2 mouse line provides a system to conditionally activate Cre recombinase in adult mouse testes upon tamoxifen treatment.

### A Clear Defect in Spermatogenesis in miR-17-92 KO Mice

Spermatogenesis is a cyclic process that consists of SSC differentiation, meiotic cell division, and the formation of haploid spermatids; the Sertoli cells, as the only somatic cells within seminiferous tubules, play a key role in providing the immediate environment for developing germ cells, to maintain the cyclic waves of spermatogenesis and fertility, it is essential for keeping a balance between SSC self-renewal and differentiation in the adult testis.^34^

**FIGURE 2:**
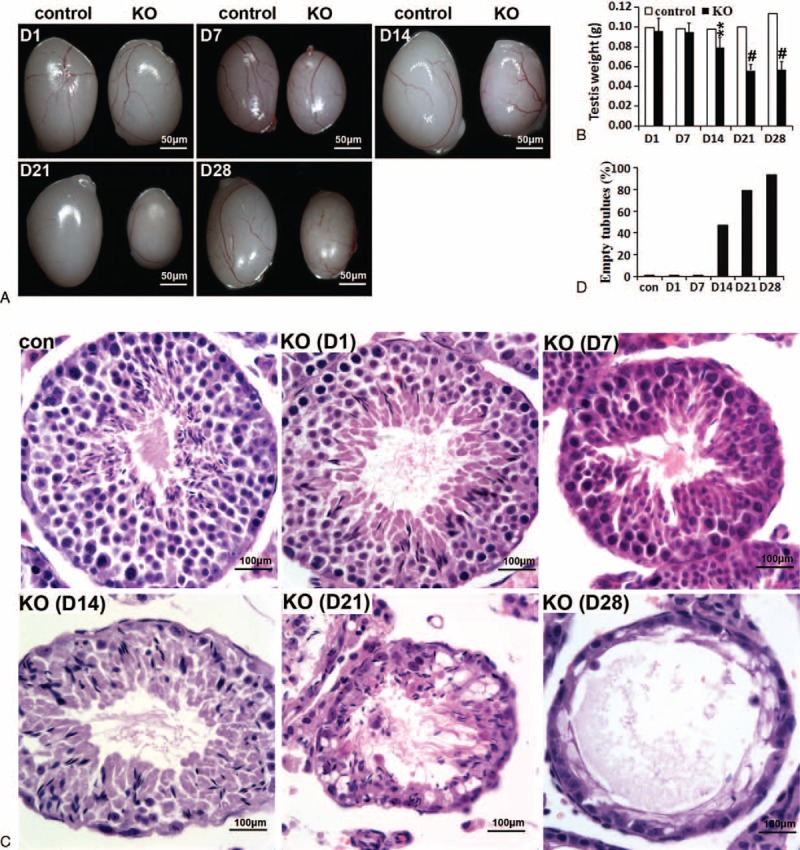
Adult mice with a targeted disruption of miR-17-92 show testis atrophy and male germ cell loss. A, Testes from control (left) and KO (right) male mice. B, Testis weight in control and KO mice (n > 5). Other details as in (F). C, Testis histological phenotype of adult control and KO mice. D, Statistical analysis of empty seminiferous tubules detected by H&E staining. D1, D7, D14, D21, and D28 represent the 1st, 7th, 14th, 21st, and 28th days after the last tamoxifen injection.

In adult mice, the spermatogenic cycle is approximately 30 days. Therefore, we selected the following 5 time points to collect samples: the 1st, 7th, 14th, 21st, and 28th day after the last tamoxifen injection (D1, D7, D14, D21, and D28). The postnatal growth of the miR-17-92 KO mice was indistinguishable from that of their littermates. Mice with a targeted disruption of miR-17-92 showed testis atrophy (Figure 2A and B). A significant difference in adult testis weight was measured at D14 and the difference in testis weight increased over time (Figure 2B). At D21 and D28, testes from adult miR-17-92 KO males on average weighed 2 times less than testes from control mice (Figure 2B). These results suggest a reduction or elimination of germ cells in mutant testes, which encouraged us to perform histological analysis.

There is no difference in testis weight and size between control and miR-17-92 KO adult mice at D1 and D7 (Figure 2A and B), but the histological analysis clearly revealed that cells at the basement membrane of seminiferous tubules, which are the normal initiation of spermatogenesis, were lost in mutant testes at D7 (Figures 2C and S2). H&E staining indicated that the germ cells were severely depleted in D14, D21, and D28 mutant testes (Figures 2C, D, S2, and S3). By D28 most seminiferous tubules were devoid of any germ cells, containing only morphologically normal Sertoli cells at the basement membrane (Figures 2C, D, and S3), which expressed GATA-4 (Figure S3), a marker of mature Sertoli cells. These findings demonstrate a clear defect in spermatogenesis in miR-17-92 KO mice.

### Reduction in Sperm Production in miR-17-92 KO Mice

**FIGURE 3:**
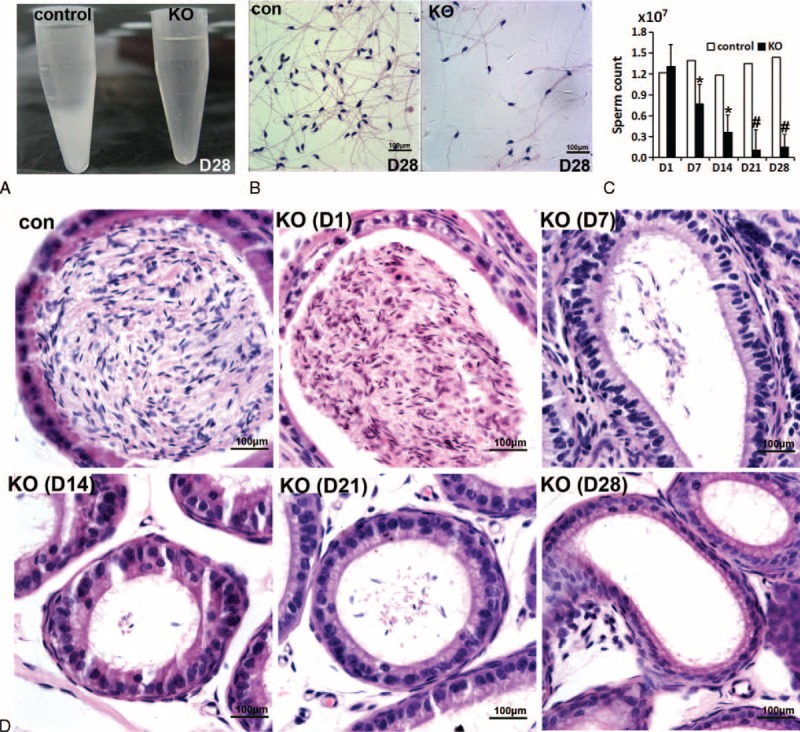
Reduced sperm production in adult miR-17-92 KO mice. A, B, Semen analysis in control and KO mice at D28. C, Number of sperm in semen in control and KO mice (n>5). D, Histological analysis of epididymis sections in control and KO mice.

To determine whether the observed testicular changes led to altered sperm production, we evaluated mature sperm collected from the vas deferens and cauda epididymis of miR-17-92 KO males and their control littermates at D1-D28. Based on the control and miR-17-92 KO mice, an analysis of sperm revealed a pretty significant reduction in sperm counts (Figure 3A–D). A significant difference in sperm number was counted as early as D7, and the difference in sperm number remarkably increased over time (Figure 3C). By D21 and D28, the epididymal spermatozoa number and sperm counts of miR-17-92 KO mice were much lower than those of controls (∼10% of control) (Figure 3C). Histological analysis of epididymides from miR-17-92 KO mice at D7–D28 showed few sperm relative to controls (Figures 3D and S4). At D14, we observed elongating spermatids (Figure S8B); however, whether the elongating spermatids can differentiate into mature sperm in the absence of the miR-17-92 cluster cannot be concluded. Moreover, our results revealed that there was no difference in the morphology and motility of mature sperms between control and miR-17-92 KO mice (Figure S8B and C). Collectively, seminiferous tubule degeneration in miR-17-92 KO mice is associated with the reduced production of mature sperms, but the part of adult miR-17-92 KO male mice remain fertile.

### Loss of Spermatogonia and SSCs in Mutant Testes

As described above, the progressive germ cell loss was observed in mutant testes, which prompted us to further determine whether miR-17-92 deletion in mouse testes led to the loss of spermatogonia and SSCs.

It is known that the SSCs and spermatogonia reside on the basement membrane of the seminiferous tubules in mammalian testes, and Apaired (Apr), Aaligned (Aal), A1, A2, A3, and A4 spermatogonia develop via a mitotic process.^35,36^

As shown in Figures 2C and S2, histological analysis of testis sections from miR-17-92 KO mice clearly revealed the germ cells along the basement membrane of seminiferous tubules of mutant testes were found to be significantly lost as early as D7, indicating that miR-17-92 deletion in mutant testes might result in the loss of spermatogonia and SSCs.

**FIGURE 4:**
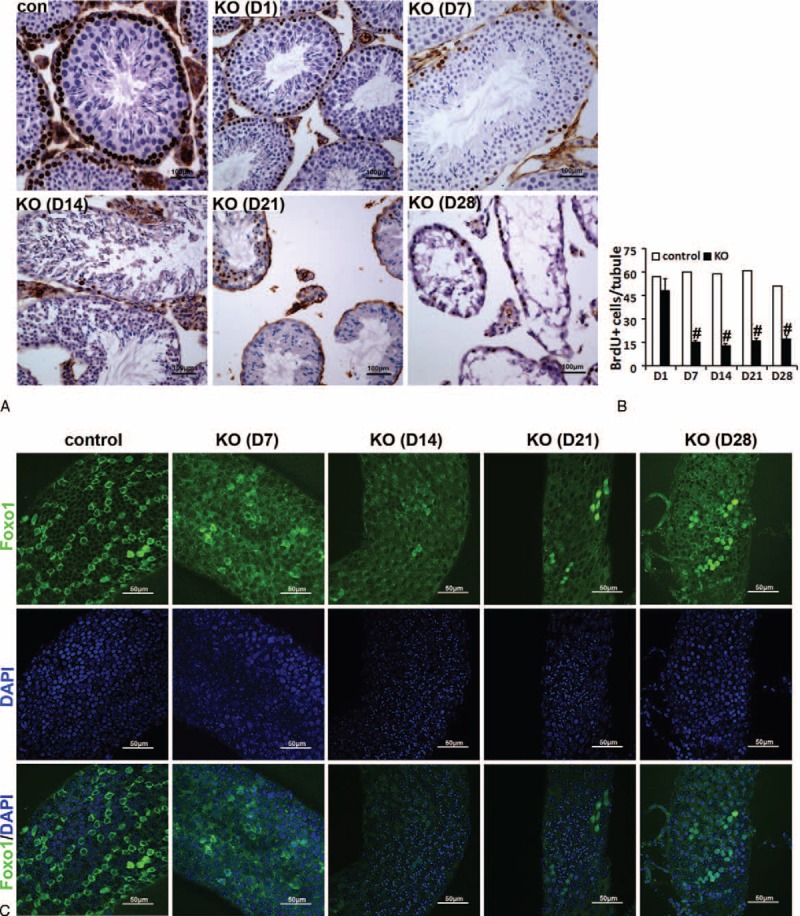
Loss of spermatogonia and spermatogonial stem cells (SSCs) in adult mutant testes. A, B, The number of BrdU-positive germ cells significantly decreases from D7 onward. C, Whole-mount immunofluorescence localization of Foxo1 in seminiferous tubules of miR-17-92 KO mice.

The mitotic cells (eg, spermatogonia and SSCs) are labeled by BrdU. Our results showed that after the continuous injection of BrdU for 6 days, 90% of seminiferous tubules of adult testes were labeled by BrdU (Figure S5A and B), and almost all germ cells (ie, spermatogonia and SSCs) at the basement membrane of BrdU-labeled seminiferous tubules can be labeled by BrdU (Figure S5A and C). More importantly, spermatogonia and SSCs only can be marked by BrdU incorporation after BrdU injection for 6 consecutive days (Figure S5A and C). In summary, the continuous injection of BrdU for 6 days can effectively label spermatogonia and SSCs within the seminiferous tubules of adult testes. BrdU incorporation revealed that compared with control testes, the number of BrdU-positive spermatogonia in mutant testes significantly decreased at D7, D14, D21, and D28 (Figure 4A and B), suggesting that miR-17-92 deletion leads to a remarkable decrease in the number of mitotic spermatogonia in mutant testes.

The previous study showed that Foxo1 is a specific marker of undifferentiated spermatogonia (As spermatogonia) in the adult testes and is required in mouse SSCs for the initiation and maintenance of spermatogenesis.^37^ To further determine the effect of miR-17-92 loss in adult testes on undifferentiated spermatogonia, we performed whole-mount immunofluorescence to localize Foxo1 in seminiferous tubules from control and mutant testes (Figures 4C and S6A). Our results indicated that compared with control, the number of Foxo1-positive cells in mutant testes significantly decreased by D7, and the decreased trend in the number of Foxo1-positive cells in D14, D21, and D28 miR-17-92 KO testes became more apparent (Figure 4C), indicating the SSC loss in adult miR-17-92 KO testes.

As described above, a balance between SSC self-renewal and differentiation in the adult testis is essential for the maintenance of cyclic waves of spermatogenesis and fertility.^34^ Therefore, this progressive loss of spermatogonia and SSCs in the seminiferous tubules of adult miR-17-92 KO mice impairs the spermatogenic process and consequently decreases the production of mature spermatozoa.

### miR-17-92 Mutant Mice Exhibit Germ Cell Apoptosis

Our above results revealed that miR-17-92 KO testes exhibited the seminiferous tubule degeneration due to germ cell massive loss. miR-17-92 has been demonstrated to play an antiapoptotic role.^5,38,39^ These findings suggest that germ cell loss in mutant testes may, at least in part, be due to apoptosis induced by miR-17-92 deletion.

**FIGURE 5:**
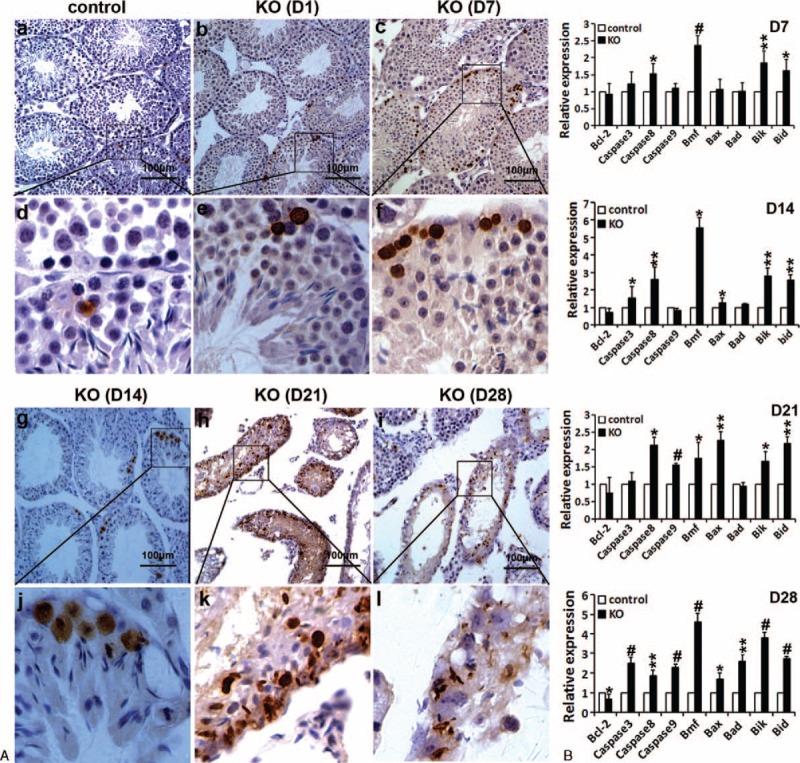
miR-17-92 ablation in adult testes results in germ cell apoptosis. A, TUNEL assay revealed the high number of apoptosis cells in mutant testes at D1, D7, D14, D21, and D28. B, qRT-PCR analysis of apoptosis-related gene expression in miR-17-92 KO testes at D7, D14, D21, and D28.

TUNEL assay clearly revealed that more apoptotic cells were detected in D7–D28 mutant testes compared with control seminiferous tubules (Figures 5A and S6B). At D7, the apoptotic cells resided mainly on the basement membrane of seminiferous tubules of mutant testes (Figure 5A), which is associated with the loss of germ cells (ie, spermatogonia) only along the basement membrane of seminiferous tubules of mutant testes at D7 (Figures 2C and S2). Apoptotic germ cells of mutant testes at D14, D21, and D28 were significantly increased (Figure 5A). Moreover, qRT-PCR analysis displayed the altered expression (upregulation or downregulation) of apoptosis-related genes (Bcl-2, Caspase 3, Caspase 8, Caspase 9, Bmf, Bax, Bad, Bik, or Bid) in D7–D28 mutant testes (Figure 5B), as confirmed by TUNEL assay (Figure 5A). Together, our findings demonstrate that germ cell apoptosis induced by miR-17-92 deletion, at least partially if not all, contributes to germ cell loss observed in mutant testes.

### Overactivation of mTOR Signaling in Mutant Testes

**FIGURE 6:**
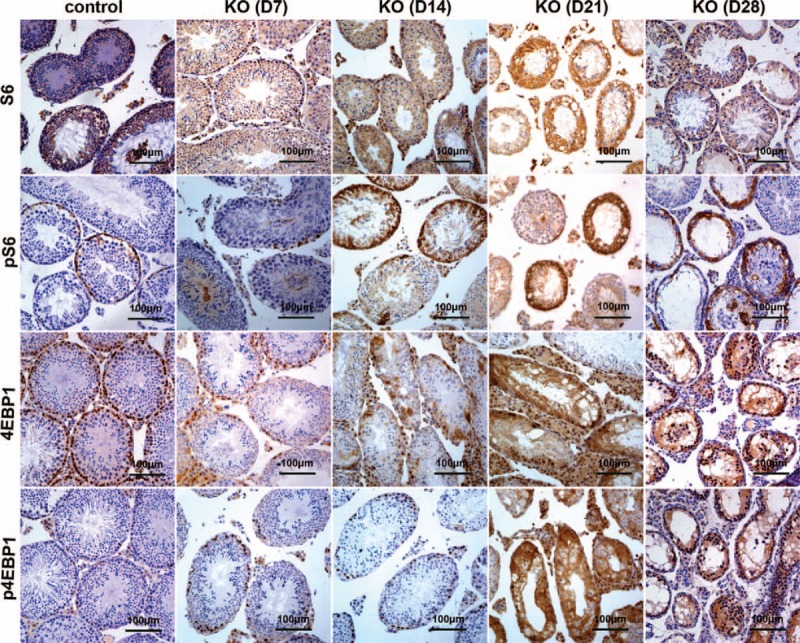
Overactivation of mTOR signaling in miR-17-92 KO testes detected by immunohistochemistry. The levels of mTOR signaling pathway components (ie, S6, pS6, 4EBP1, and p4EBP1) in adult testes of miR-17-92 KO mice at D7, D14, D21, and D28 were detected by the immunohistochemistry.

**FIGURE 7:**
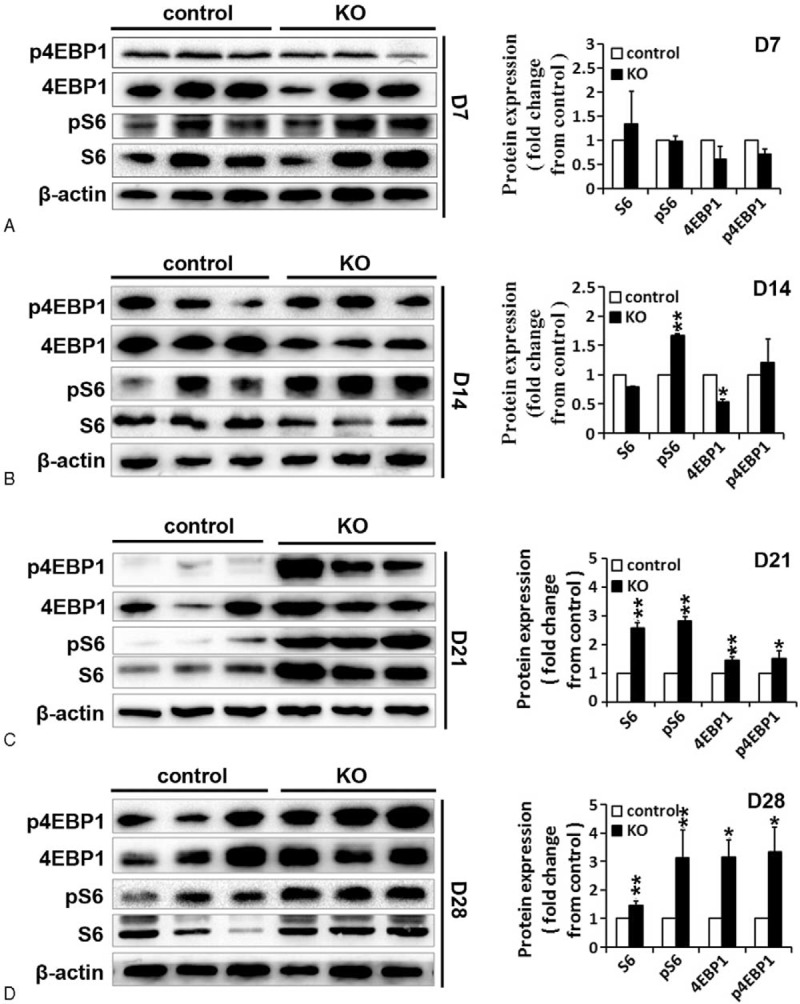
Overactivation of mTOR AQ1 signaling in miR-17-92 KO testes detected by Western blot. Western blot analysis and quantification (n=3 per group) of S6, pS6, 4EBP1, and p4EBP1 in adult testes of miR-17-92 KO mice at D7 (A), D14 (B), D21 (C), and D28 (D) were performed. β-actin was used as a loading control.

The previous study indicates that translational regulation is differentially dependent on the mTOR (mammalian target of rapamycin) signaling pathway in mouse spermatocytes and spermatids.^40^ Overactivation of mTOR signaling in mouse testes impaired spermatogenesis.^41^ miR-17-92 has been demonstrated to be involved in the regulation of mTOR pathway.^42^ Therefore, we analyzed the expression of mTOR pathway members (ie, S6, pS6, 4EBP1, and p4EBP1) by IHC (Figures 6 and S6C) and Western blot (Figure 7) to better understand the mechanisms involved in the development of the testicular phenotypes observed in miR-17-92 KO testes. IHC and Western blot analysis showed the increased expression of S6, pS6, 4EBP1, and p4EBP1 in mutant testes compared with controls (Figures 6 and 7). At D7, the results from IHC and Western blot indicate that there is no difference in the expression of p4EBP1, pS6, 4EBP1, and S6 between control and mutant testes (Figures 6 and 7A). The increased level of phosphorylated S6 was observed in D14 mutant testes (Figures 6 and 7B), while at D21 and D28, the significantly enhanced expression of S6, pS6, 4EBP1, and p4EBP1 was observed in mutant testes compared with controls (Figures 6 and 7C, D). Taken together, our data demonstrate the increased activation of mTOR signaling in miR-17-92 KO testes, suggesting that the overactivation of mTOR signaling in mutant testes might contribute to the damaged spermatogenesis in miR-17-92 KO mice.

### Identification of miR-17-92 Targets Involved in Testicular Phenotypes Observed in miR-17-92 KO Testes

**FIGURE 8:**
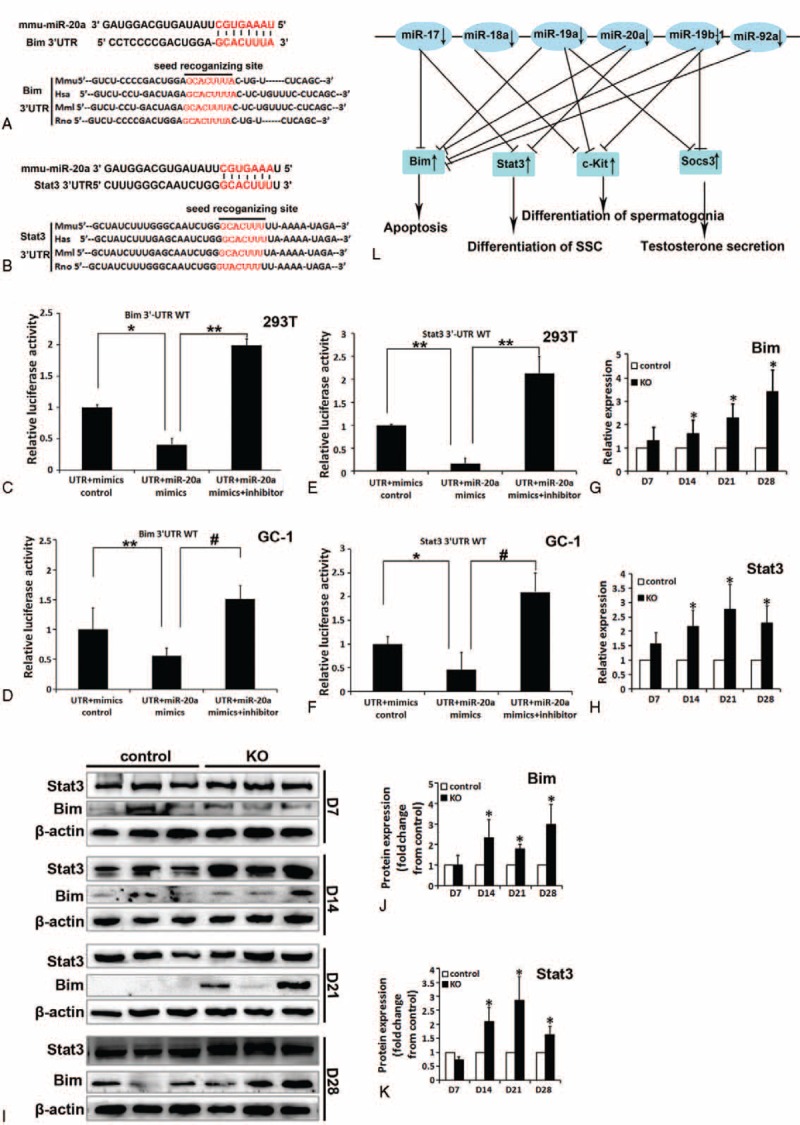
Identification of Bim and Stat3 as target genes of miR-20a. A, B, Sequence alignment of 3’-UTR of human (Hsa), mouse (Mmu), rhesus (Mml), and rat (Rno) Bim (A) and Stat3 (B) highlighting miR-20a binding site. C–F, Both Bim (C, D) and Stat3 (E, F) are target genes of miR-20a. The luciferase reporter assay in GC-1 and 293T cells was performed as described in the Methods section. G, H, qRT-PCR analysis of Bim (G) and Stat3 (H) expression in miR-17-92 KO testes. I–K, Western blot analysis of Bim (I, J) and Stat3 (I, K) expression in miR-17-92 KO testes. L, A proposed model on the roles of the miR-17-92 cluster in mouse spermatogenesis by coordinated regulation of multiple genes.

Subsequently, we sought to further explore the direct molecular mechanisms underlying such pleiotropic effects of miR-17-92 deletion in mouse testis. It is generally accepted that miRNAs exert their functions by regulating expression of their downstream target gene(s). Therefore, putative targets of the members of the miR-17-92 cluster involved in these above-mentioned functions of miR-17-92 in mouse testis were predicted by using common databases, such as microRNA.org, RNAhybrid, and miRWalk. Among these experimentally verified or bioinformatically predicted target genes of miR-17-92 cluster members, Bim/Bcl2l11, Stat3, c-Kit, and Socs3 (suppressor of cytokine signaling 3) (Figure 8L) especially caught our attention. The key reasons are as follows: Bim and Stat3 genes harbor miR-20a binding sites, and c-Kit and Socs3 genes harbor miR-19 binding sites, which are conserved across different phyla (ie, human, monkey, mouse, and rat) (Figures 8A, B and S7A, B); Bim is identified as direct targets of miR-17,^43,44^ miR-20a,^44^ and miR-92a;^44^ Stat3 is identified as direct targets of miR-17^45,46^ and miR-20a;^45,46^ Socs3 is identified as a direct target of miR-19a;^47^ and Bim, Stat3, c-Kit, and Socs3 have been demonstrated to be implicated in the process of spermatogenesis.^48–54^

The 3’-UTRs of Bim and Stat3 mRNA contain complementary site for the seed region of miR-20a (Figure 8A and B), and the 3’-UTRs of c-Kit and Socs3 mRNA contain complementary site for the seed region of miR-19 (data not shown). We generated reporter constructs in which the luciferase coding sequence was fused to the 3′-UTRs of these genes. Measurements of luciferase activity in 293T or GC-1 cells transfected with miR-20a mimics plus reporter plasmid containing the wild-type 3’-UTRs of Bim or Stat3 exhibited a significant reduction of luciferase activity (Figure 8C–F), respectively. In contrast, transient transfection of wild-type Bim-luc reporter or Stat3-luc reporter with miR-20a mimics plus inhibitor into 293T or GC-1 cells could fully reverse the miR-20a-induced decrease in luciferase activity (Figure 8C–F). To examine the influences of miR-17-92 deletion on endogenous expression of these miR-17-92 targets (ie, Bim, Stat3, c-Kit, and Socs3), we first determined their expression in miR-17-92 mutant testes. qRT-PCR and Western blot analysis revealed the increased expression of Bim, Stat3, c-Kit, and Socs3 in D14, D21, and D28 mutant testes, compared with control testes (Figures 8G–K and S7C–H). Moreover, a previous study showed that overexpression of miR-17-92 cluster in spermatogonia led to a significant reduction in the expression of Bim, Stat3, c-Kit, and Socs3.^16^ Therefore, miR-17-92 negatively regulates the expression of Bim, Stat3, c-Kit, and Socs3 in mouse testes. More importantly, these aforementioned results suggest that Bim, Stat3, c-Kit, and Socs3 are direct targets of the members of miR-17-92 cluster.

## DISCUSSION

There is abundant evidence for the roles of miR-17-92 cluster in cancer.^5,15^ However, very little is known about the functions of this cluster in spermatogenesis. The present study demonstrates a clear defect in spermatogenesis in miR-17-92 KO mice, as shown by testicular atrophy, germ cell loss, and reduced sperm production (Figures 2 and 3).

Despite the severe loss of male germ cells in miR-17-92 KO testes, miR-17-92 KO males are fertile (data not shown). One scenario is provided to explain this observation. Because the efficiency of Cre-mediated excision in mice does not reach 100%, miR-17-92 cluster cannot be completely deleted in all germ cells. Actually, some normal seminiferous tubules with multiple layers of germ cells were observed in miR-17-92 KO testes (Figure S8A). Type A spermatogonia in these normal tubules can produce into mature sperms, and the adult miR-17-92 KO male mice are fertile.

A previous in vitro study revealed that miR-17-92 is highly expressed in THY1^+^-enriched undifferentiated spermatogonia, and the members of miR-17-92 cluster are remarkably downregulated during in vitro retinoic acid (RA)-induced spermatogonial differentiation.^15^ miR-17-92 has been demonstrated to positively regulate the self-renewal and proliferation of cancer stem cells,^55,56^ hematopoietic and neural stem cells.^57–59^ These findings suggest that miR-17-92 cluster may play important roles in SSC self-renewal and proliferation of the undifferentiated spermatogonia.

Our results indicated that among germ cells in D7 mutant testes, only spermatogonia and SSCs were clearly found to be lost, and subsequently loss of germ cells, including spermatogonia and SSCs, became more apparent in D14, D21, and D28 miR-17-92-mutant testes (Figures 2 and 3). Furthermore, at D7, the apoptotic germ cells were found to reside mainly on the basement membrane of seminiferous tubules of mutant testes (Figure 5A). The previous study revealed that about 80 genes (including Bim, Stat3, c-Kit, and Socs3) upregulated upon RA-induced spermatogonial differentiation were potentially targeted by the members of miR-17-92 cluster,^15^ as experimentally confirmed by this study (Figures 8 and S7) and other previous studies.^43–47^ Of these, Bim, a BH3-only protein, is known to be a proapoptotic gene,^52^ loss of Bim inhibited male germ cell apoptosis in bcl-x-deficient mice,^51^ and Bim and Bax cooperate to initiate apoptosis of surplus spermatogonia since Bax^−/−^/Bim^−/−^ caused an accumulation of spermatogonia in testes.^48^ Stat3 has been demonstrated to promote the differentiation of SSCs.^49,54^ c-Kit is a marker for differentiating spermatogonia, and positively regulates the differentiation of spermatogonia in mice.^53,60^ Socs3 is related to testosterone secretion.^50^ Additionally, miR-17-92 plays an antiapoptotic role.^5,38,39^ Our results illustrated the significantly increased expression of the target genes (ie, Bim, Stat3, c-Kit, and Socs3) of miR-17-92 cluster members in miR-17-92 KO testes, whereas miR-17-92 overexpression in spermatogonia resulted in the reduced expression of Bim, Stat3, c-Kit, and Socs3.^16^ The above data strongly support the hypothesis that these differentiation-associated genes (eg, Stat3 and c-Kit) are repressed by miR-17-92 in undifferentiated spermatogonia, and miR-17-92 cluster deletion in mouse testes promotes the differentiation of spermatogonia and SSCs by inducing the expression of differentiation-associated genes (eg, Stat3 and c-Kit). Moreover, these aforementioned data also strongly support another hypothesis that these proapoptosis-associated genes (eg, Bim) are repressed by miR-17-92 in spermatogonia, and miR-17-92 cluster deletion in mouse testes promotes the apoptosis of spermatogonia and SSCs by upregulating the expression of proapoptosis-associated genes (eg, Bim). Taken together, all these aforementioned results suggest that the upregulated expression of the targets (ie, Bim, Stat3, c-Kit, and Socs3) of miR-17-92 cluster members in miR-17-92-deficient testes is likely at least partially responsible for the testicular phenotypes (eg, testicular atrophy, spermatogonia and SSC loss, germ cell apoptosis, and decreased sperm production, etc) observed in mutant testes, thereby resulting in the impaired spermatogenesis in miR-17-92 KO mice (Figure 8L), which remains to be further investigated in the future.

The deregulation of mTOR signaling is often observed in aging, metabolism, and cancer.^61,62^ mTOR interacts with several proteins to form 2 distinct complexes named mTOR complex 1 (mTORC1) and 2 (mTORC2). The mTOR-containing complexes have different sensitivities to rapamycin as well as upstream inputs and downstream outputs.^63^ The molecular functions of mTOR signaling are extremely complex. Altered LKB1/AMPK/TSC1/TSC2/mTOR signaling in mouse testes caused the disruption of Sertoli cell polarity and spermatogenesis, and the overactivation of mTOR signaling was found in Lkb1^cko^, Tsc1^cko^, and Tsc2^cko^ testes.^41^ In this study, we found that the increased activation of mTOR signaling in miR-17-92 KO testes (Figures 6, S6C, and 7) might be involved in the damaged spermatogenesis. In summary, these findings suggest that the dysregulated mTOR signaling also contributes to the pathogenesis of testicular anomalies.

In the present study, we observed that the deletion of the entire miR-17-92 cluster in mouse testes caused the impaired spermatogenesis. The miR-17-92 gene cluster encodes 6 miRNAs of 4 miRNA families: the miR-17 family including miR-17-5p and miR-20a, the miR-18 family (miR-18a), the miR-19 family (miR-19a and miR-19b-1), and the miR-92 family.^5,15^ Thus, we will intend to further examine the effects of each member of miR-17-92 gene cluster in the entire process of spermatogenesis in the future. The 6 new miR-17-92-mutant mouse strains^64^ will facilitate us to further investigate the complex developmental functions of this cluster in mouse spermatogenesis.

In conclusion, our findings demonstrate that miR-17-92 is essential for normal spermatogenesis in mice. The progressive loss of spermatogonia and SSCs in the seminiferous tubules of adult miR-17-92 KO mice impairs the spermatogenic process and consequently decreases the production of mature spermatozoa.

## Supplementary Material

Supplemental Digital Content
