# Supplementary material for: Targeted Disruption of miR-17-92 Impairs Mouse Spermatogenesis by Activating mTOR Signaling Pathway
Source: Medicine (Baltimore). 2016 Feb 18;95(7):e2713. doi: 10.1097/MD.0000000000002713 (PMC4998608; doi:10.1097/MD.0000000000002713)
Supplement: Supplemental Digital Content [file medi-95-e2713-s001.doc]

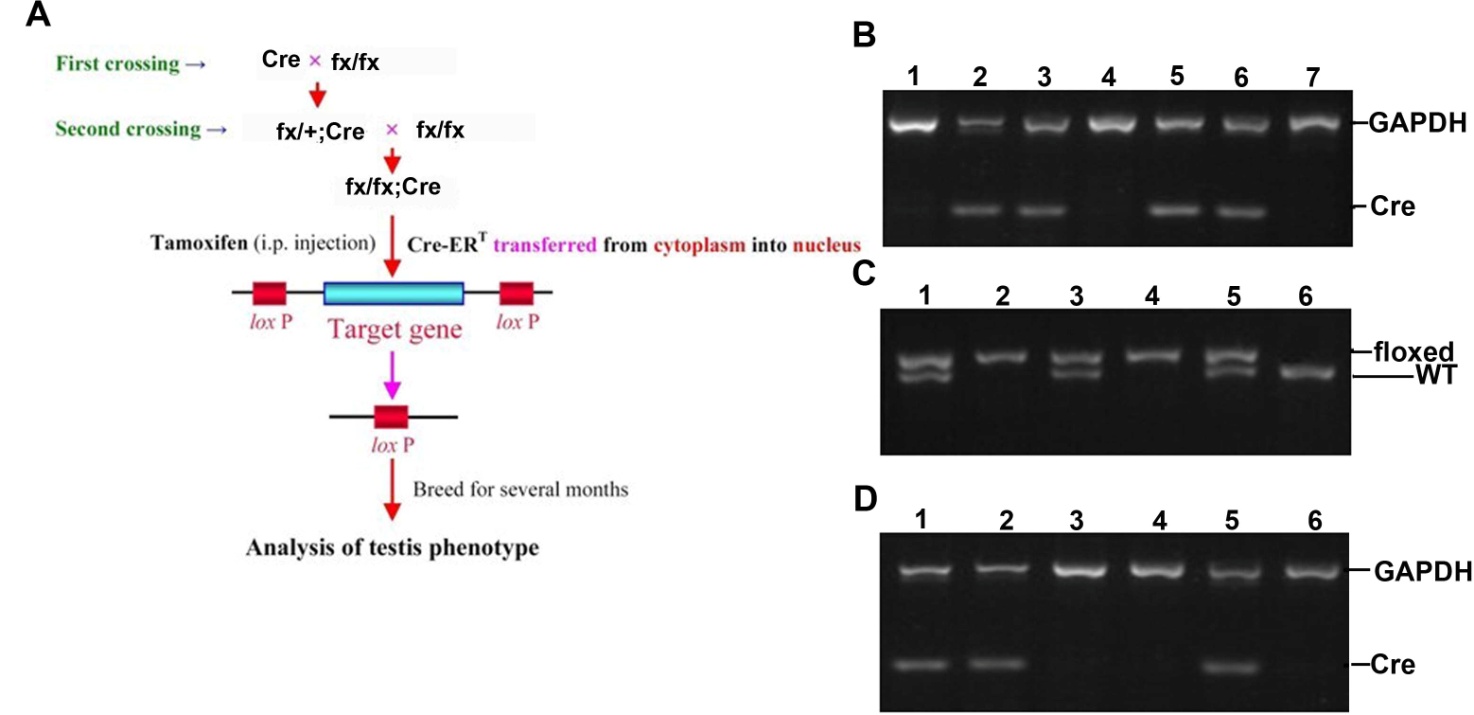


**Figure S1. Generation of miR-17-92 conditional knockout mice.**

(**A**) Strategy for generating miR-17-92^ﬂ/ﬂ^;Cre-ERT2 mice by two rounds of mating.

hUb-Cre-ERT2 mice were generated through lentitransgenesis using a lentivirus that expresses the Cre-ERT2 from the human ubiquitin C promoter.^21^ During the first crossing,

miR-17-92^ﬂ/+^;Cre-ERT2 mice (genotype: fx/+;Cre) were obtained by mating between miR-17-92^fl/fl^ mice and hUb-Cre-ERT2 mice. During the second crossing, miR-17-92^ﬂ/ﬂ^;Cre-ERT2 mice (genotype: fx/fx;Cre) were obtained by mating between miR-17-92^ﬂ/+^;Cre-ERT2 mice and miR-17-92^fl/fl^ mice.

(**B**) PCR-based Cre genotyping based on tail DNA from offspring after the first crossing. Lane 2, 3, 5 and 6: Cre-positive mice; Lane 1 and 4: Cre-negative mice; Lane 7: wild-type mouse.

(**C-D**) PCR-based genotyping for the floxed miR-17-92 mice and Cre gene based on tail DNA derived from offspring after the second crossing. Lane 2: fx/fx;Cre; Lane1 and Lane 5: fx/+;Cre; Lane 3: fx/+; Lane 4: fx/fx. Lane 6: wild type.


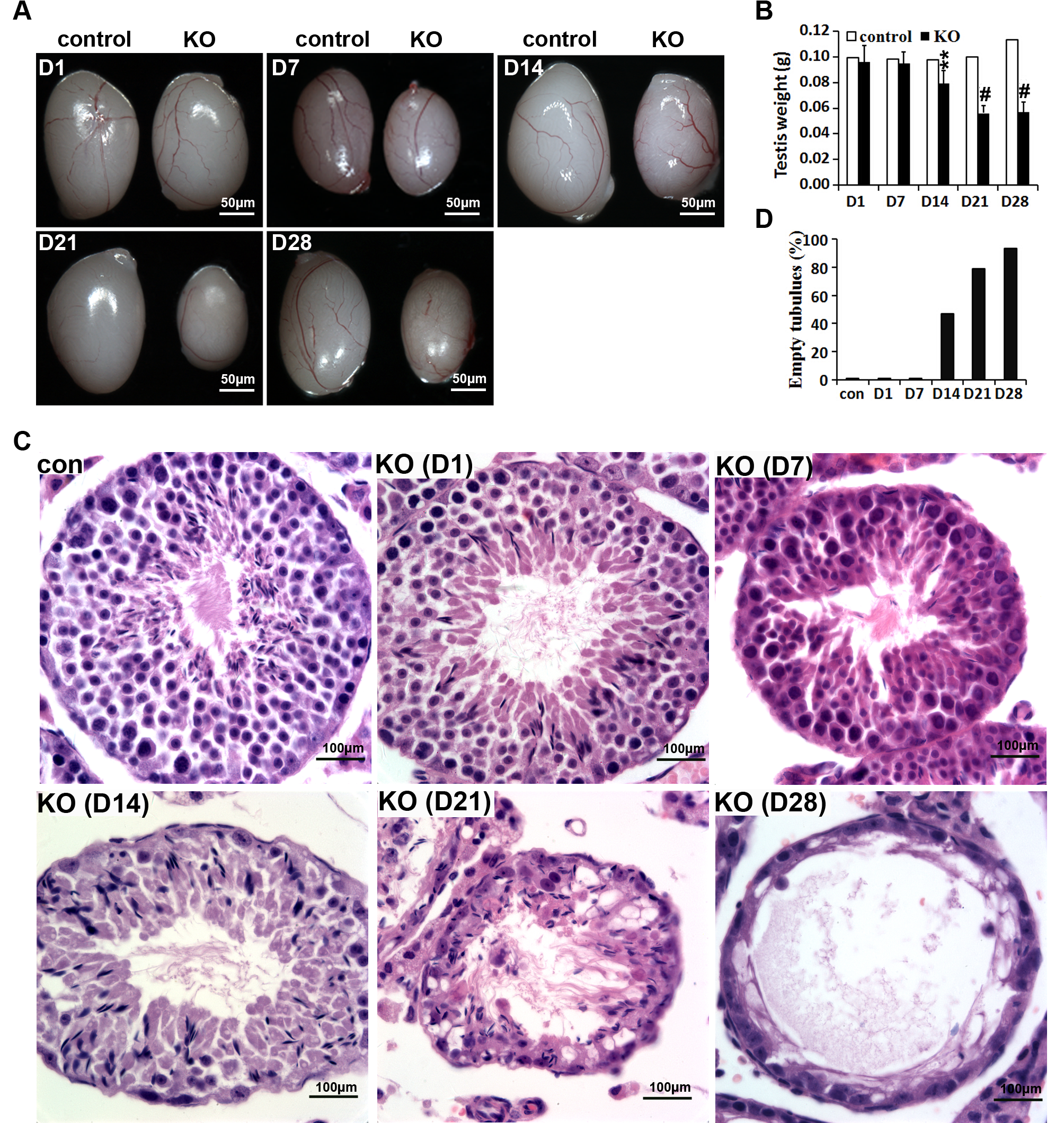


**Figure S2.** **Testis histological phenotype of adult miR-17-92 KO mice.**

**
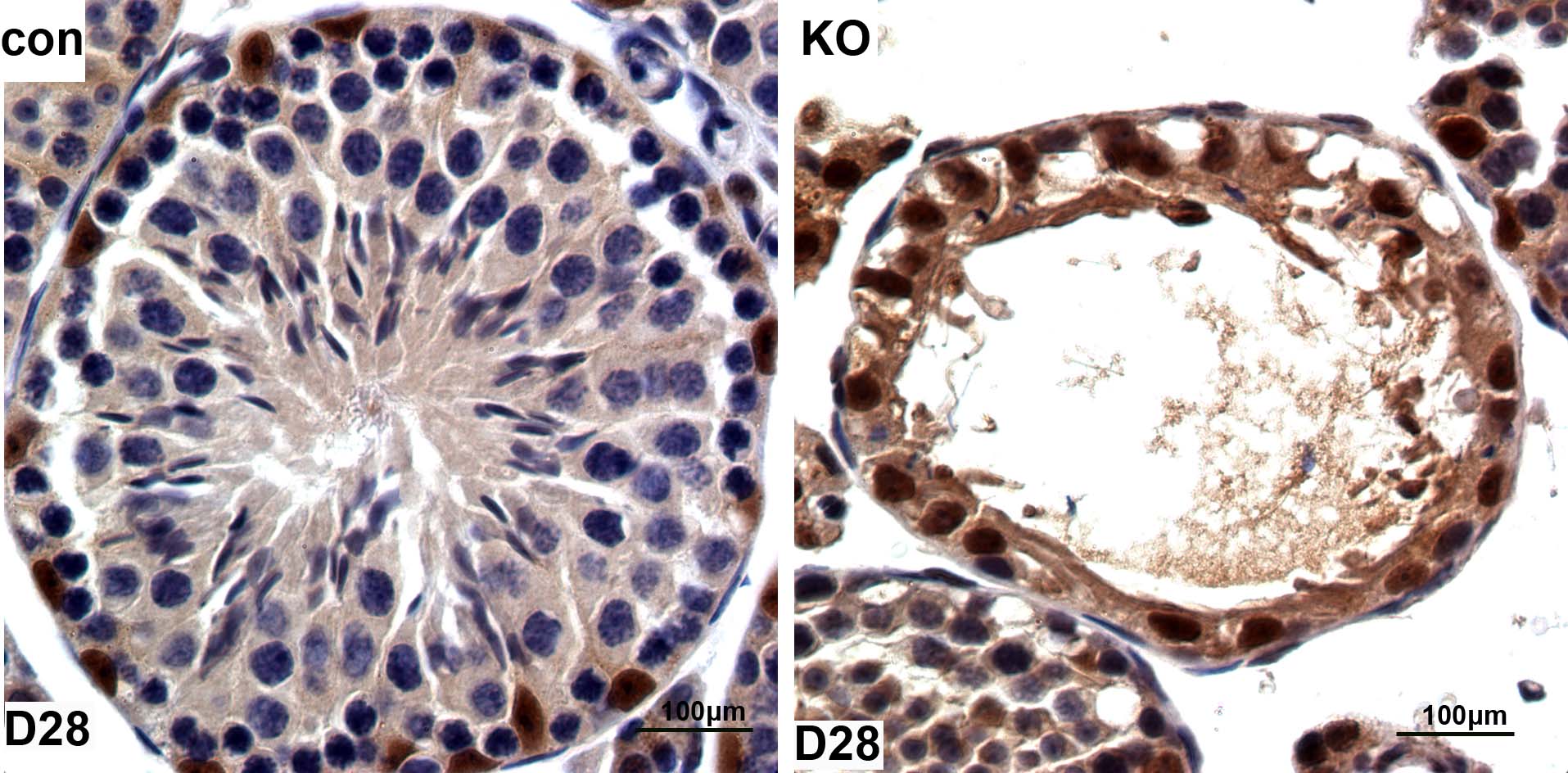
**

**Figure S3. Immunolocalization of GATA-4 in Sertoli cell nuclei at D28.**

**
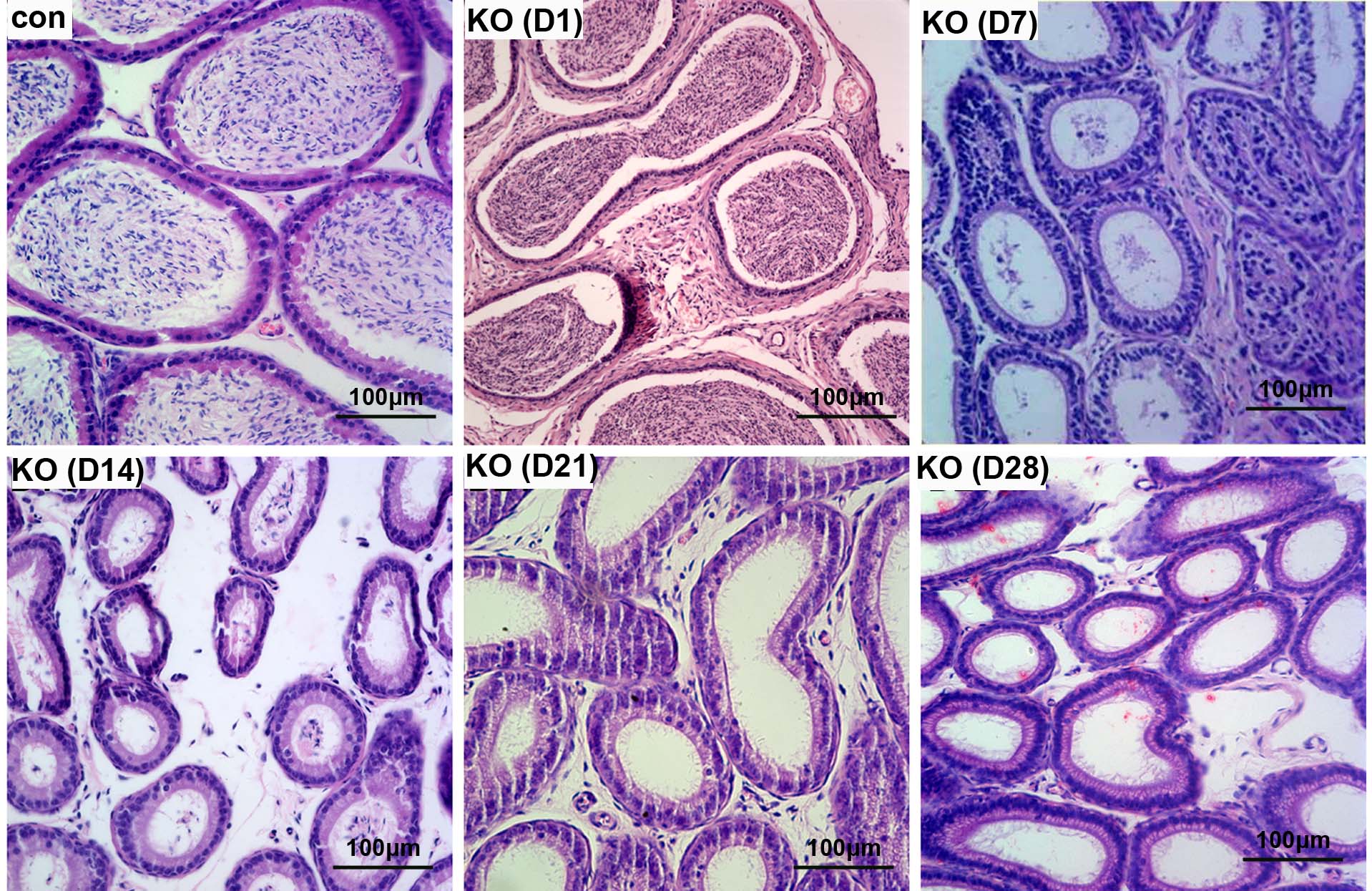
**

**Figure S4. Histological analysis of epididymis sections in control and KO mice.**


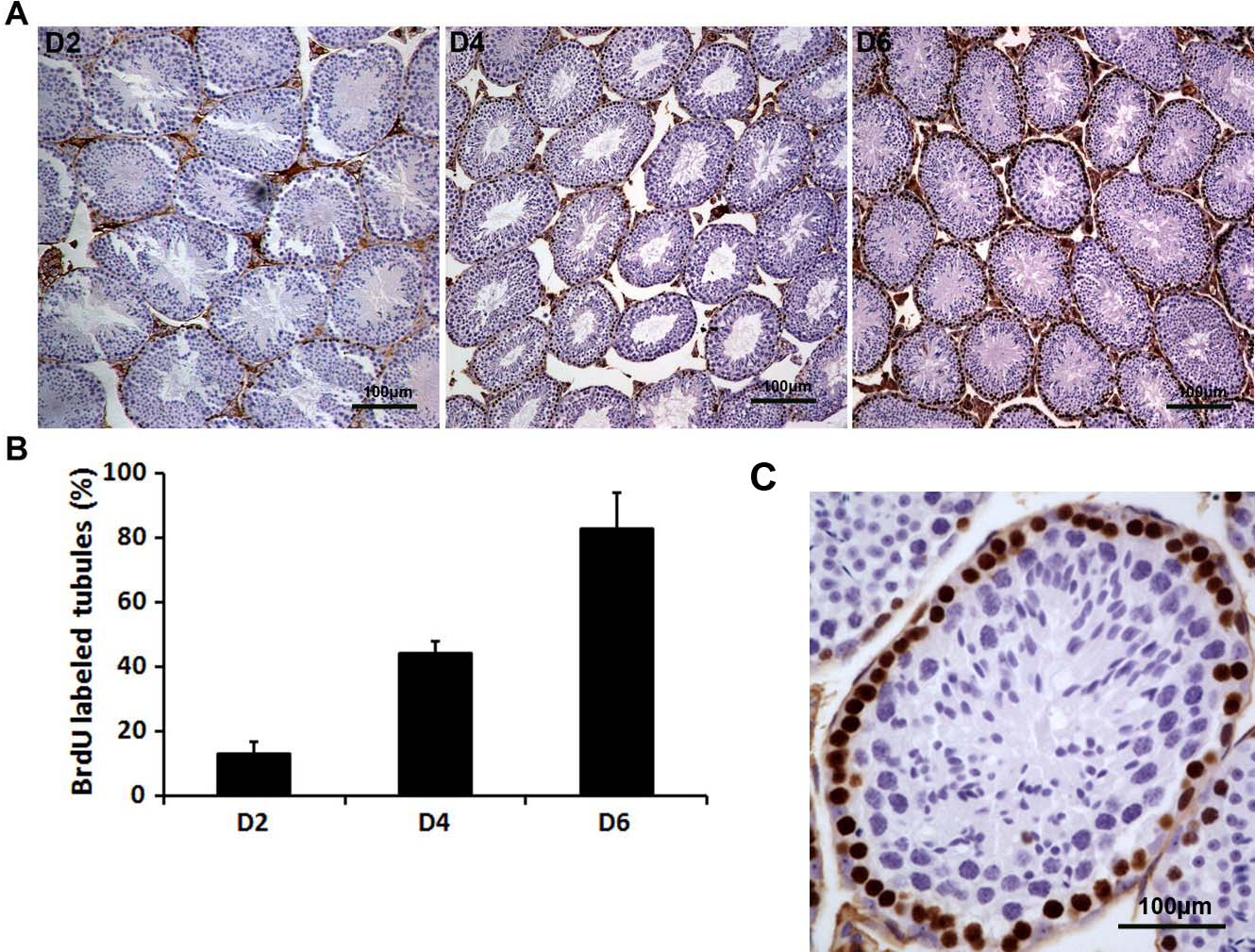


**Figure S5. Almost all spermatogonia can labeled by BrdU** **when BrdU is intraperitoneally** **injected for six consecutive days.**

(**A-B**) The situation of seminiferous tubules labeled by BrdU after the continuous injection of BrdU for 2, 4 or 6 days.

(**C**) BrdU-labeled cells are mainly located in the basement membrane after BrdU injection for six consecutive days.


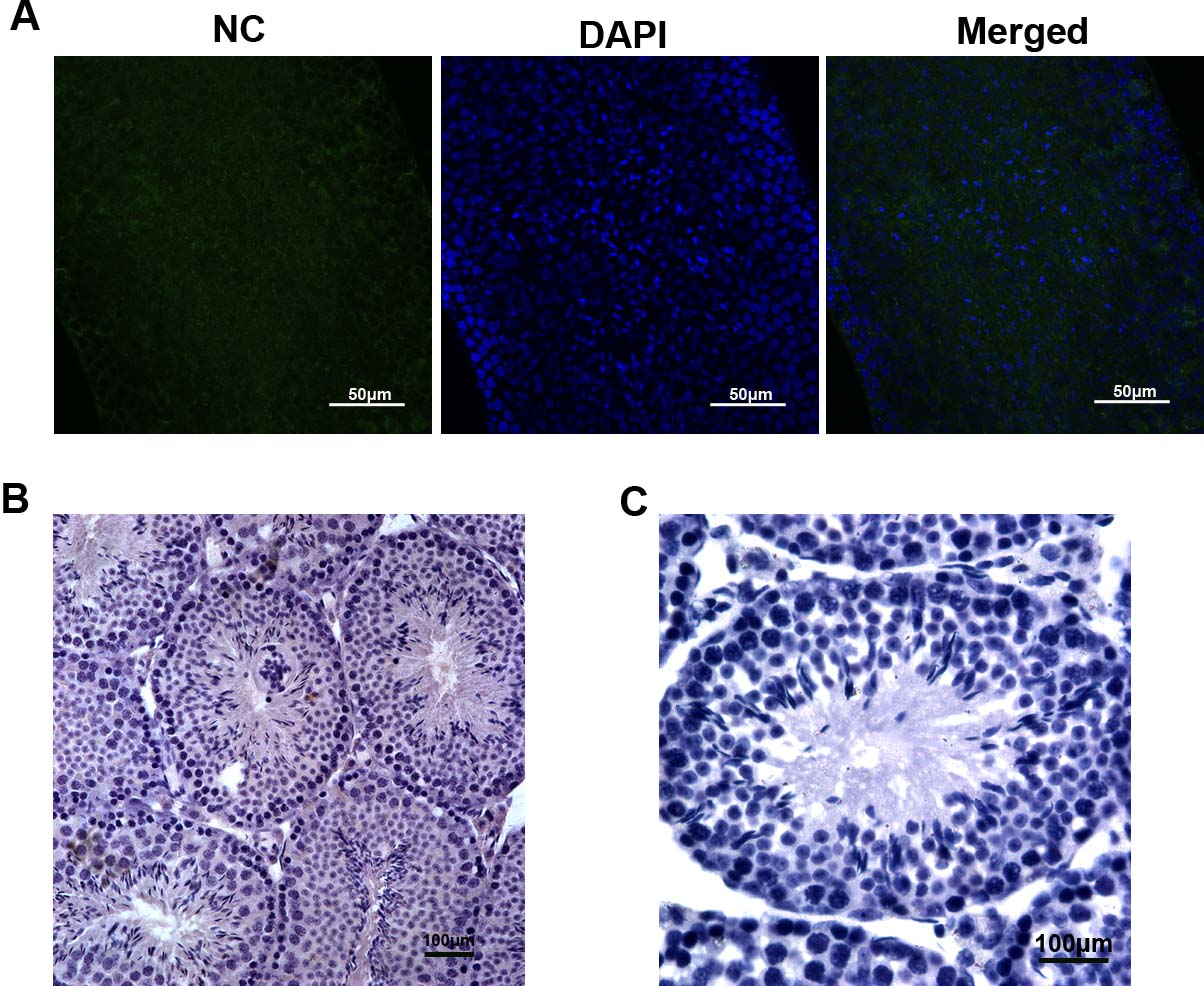


**Figure S6. Negative control (NC) for whole-mount staining, TUNEL assay and immunohistochemistry (IHC).**

(**A**) Negative control for whole-mount staining (Figures 4C).

(**B**) Negative control for TUNEL assay (Figures 5A).

(**C**) Negative control for IHC (Figures 6).


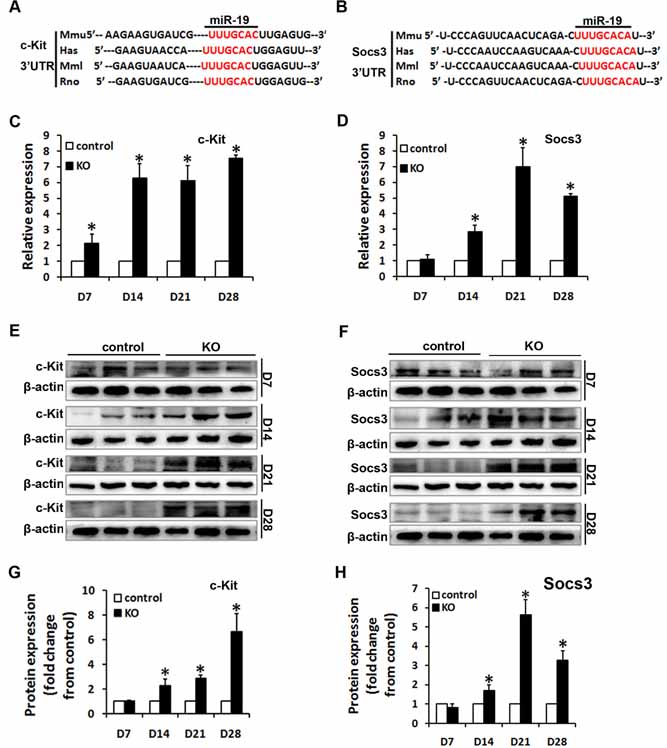


**Figure S7. Identification of C-Kit and Socs3 as target genes of miR-19.**

**(A-B)** Sequence alignment of the 3’-UTR of human (Hsa), mouse (Mmu), rhesus (Mml) and rat (Rno) c-Kit (A) and Socs3 (B) highlighting miR-19 binding site.

(**C-D**) qRT-PCR analysis of c-Kit (C) and Socs3 (D) expression in miR-17-92 KO testes.

(**E-H**) Western blots and quantification of c-Kit (E,G) and Socs3 (F,H) in miR-17-92 KO testes.

**
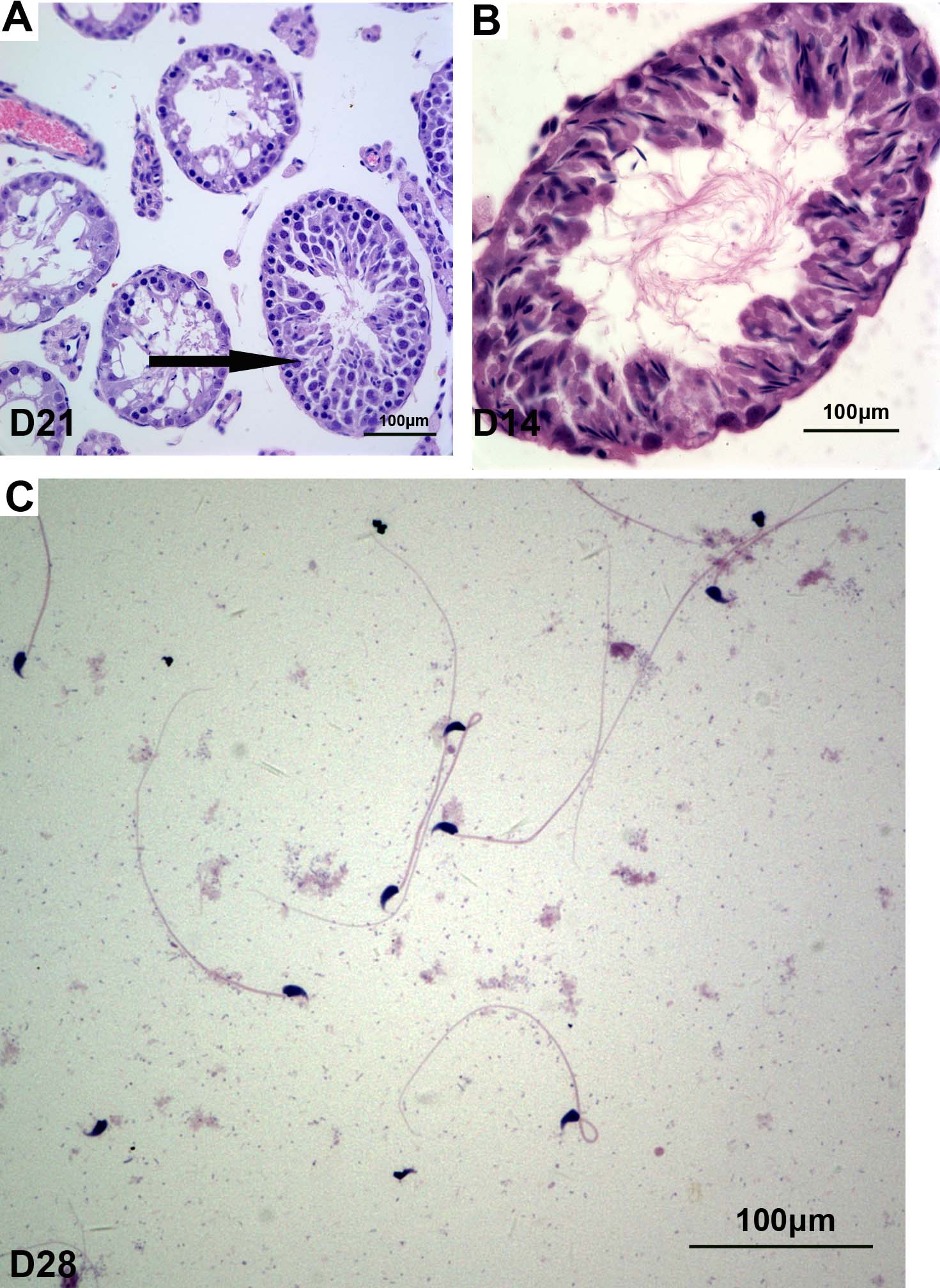
**

**Figure S8. Male mice are fertile when miR-17-92 is deleted in adult mice.**

(**A**) Normal seminiferous tubule is shown by arrowhead.

(**B**) Elongating spermatids appear in seminiferous tubule of miR-17-92 KO mice at D14.

(**C**) Sperm motility and morphology are normal in miR-17-92 KO mice.

**Table S1 Primer pairs s for PCR-based genotyping**

| **Primer name** | **Primer sequence** |
| --- | --- |
| miR-17-92 forward primer(P1) | TCGAGTATCTGACAATGTGG |
| miR-17-92 reverse primer(P2) | ATAGCCTGA AACCAACTGTGC |
| miR-17-92 reverse primer(P3) | TAGCCAGAAGTTCCA AAT TGG |
| Cre forward primer | GCCGCGCGAGATATGG |
| Cre reverse primer | GCCACCAGCTTGCATGATC |
| GAPDH forward primer | CTA GGCCACAGAATTGAAAGATCT |
| GAPDH reverse primer | GTAGGTGGA AATTCTAGCATCATCC |

**Table S2 Primer pairs**

**for qRT-PCR analysis of the individual components of miR-17-92**

| **Primer name** | **Primer sequence** |
| --- | --- |
| mmiR-17-5p RT primer | GTCGTATCCAGTGCAGGGTCCGAGGTATTCGCACTGGATACGACctacc |
| mmiR-17-5p forward primer | CAAAGTGCTTACAGTGCAG |
| mmiR-18a-5p RT primer | GTCGTATCCAGTGCAGGGTCCGAGGTATTCGCACTGGATACGACctatc |
| mmiR-18a-5p forward primer | TAAGGTGCATCTAGTGCAG |
| mmiR-19a-5p RT primer | GTCGTATCCAGTGCAGGGTCCGAGGTATTCGCACTGGATACGACgtagt |
| mmiR-19a-5p forward primer | CTGTAGTTTTGCATAGTTGCA |
| mmiR-20a-5p RT primer | GTCGTATCCAGTGCAGGGTCCGAGGTATTCGCACTGGATACGACctacc |
| mmiR-20a-5p forward primer | TAAAGTGCTTATAGTGCAG |
| mmiR-19b-1-5p RT primer | GTCGTATCCAGTGCAGGGTCCGAGGTATTCGCACTGGATACGACgctgg |
| mmiR-19b-1-5p forward primer | AGTTTTGCAGGTTTGCATC |
| mmiR-92a-1-5p RT primer | GTCGTATCCAGTGCAGGGTCCGAGGTATTCGCACTGGATACGACagcat |
| mmiR-92a-1-5p forward primer | AGGTTGGGATTTGTCGCAA |
| universal reverse primer | GTGCAGGGTCCGAGGTATTC |
| RNU6-1 RT primer | AACGCTTCACGAATTTGCGT |
| RNU6-1 forward primer | CTCGCTTCGGCAGCACA |
| RNU6-1 reverse primer | AACGCTTCACGAATTTGCGT |

**Table S3 Primer pairs for qRT-PCR analysis of apoptosis-related gene**

| **Gene** | **Forward primer (5’-3’)** | **Reverse primer (5’-3’)** |
| --- | --- | --- |
| Bcl-2 | GAGGATTGTGGCCTTCTTT | CGTTATCCTGGATCCAGGTG |
| Caspase3 | GCAGGAGACCATCGAGGATG | GACTGGATGAACCACGACCC |
| Caspase8 | GGGTGCTGTCTATGGGACAG | CACCATCTCCTCTCGGTTGC |
| Caspase9 | GCTGAGACGCTGTCCTGGAG | GAGGAAGTGCAGGCCACCTC |
| Bmf | GTGAGCGGCTGCTTGTCTGG | CAAACAGGTCAGCAGAGAGC |
| Bax | GCGATGAGATGGACCACAAC | CTTCCAGATGGTGAGCGAGG |
| Bad | CCTGCATCGGCGATGAGATG | CTCTGGGGAAGGCTGTCTTC |
| Bik | GCGATGAGATGGACCACAAC | CTCTGACACCTGTCCGGCTG |
| Bid | GTGGGCCGCTCTAGGCACCAA | CTCTGGGGAAGGCTGTCTTC |
| β-actin | GTGGGCCGCTCTAGGCACCAA | CTCTTTGATGTCACGCACGATTTC |

**Table S4 Primer pairs for qRT-PCR analysis of the indicated genes**

| **Gene** | **Forward primer (5’-3’)** | **Reverse primer (5’-3’)** |
| --- | --- | --- |
| Bim | CTCCCTACAGACAGAACCGC | CGTTGAACTCGTCTCCGATC |
| Stat3 | CAATACCATTGACCTGCCGAT | GAGCGACTCAAACTGCCCT |
| c-Kit | GCCACGTCTCAGCCATCTG | GTCGCCAGCTTCAACTATTAACT |
| Socs3 | ATGGTCACCCACAGCAAGTTT | TCCAGTAGAATCCGCTCTCCT |

**Table S5 List of antibodies and suppliers used for immunoblotting analysis**

| **Antibody** | **Source** | **Suppliers** |
| --- | --- | --- |
| S6 | Rabbit | Cell Signaling Technology |
| pS6 | Rabbit | Cell Signaling Technology |
| 4EBP1 | Rabbit | Cell Signaling Technology |
| p4EBP1 | Rabbit | Cell Signaling Technology |
| Bim | Rabbit | Bioworld Technology |
| Stat3 | Mouse | Cell Signaling Technology |
| BrdU | Mouse | GE Healthcare |
| Foxo1 | Rabbit | Cell Signaling Technology |
| β-actin | Rabbit | Bioss |
